# Reactive oxygen species levels control NF-κB activation by low dose deferasirox in erythroid progenitors of low risk myelodysplastic syndromes

**DOI:** 10.18632/oncotarget.22299

**Published:** 2017-11-06

**Authors:** Mathieu Meunier, Sarah Ancelet, Christine Lefebvre, Josiane Arnaud, Catherine Garrel, Mylène Pezet, Yan Wang, Patrice Faure, Gautier Szymanski, Nicolas Duployez, Claude Preudhomme, Denis Biard, Benoit Polack, Jean-Yves Cahn, Jean Marc Moulis, Sophie Park

**Affiliations:** ^1^ CHU Grenoble Alpes, University Clinic of Hematology, Grenoble, France; ^2^ Université Grenoble Alpes, CNRS UMR 5525, Grenoble INP, CHU Grenoble Alpes, TIMC-IMAG ThEREx, Grenoble, France; ^3^ Laboratory of Hematology, CHU Grenoble Alpes, Grenoble, France; ^4^ Unité de Biochimie Hormonale et Nutritionnelle, Département de Biologie - Toxicologie - Pharmacologie, CHU Grenoble Alpes, Grenoble, France; ^5^ Plateforme de Microscopie Photonique - Cytométrie en Flux, Institut Albert Bonniot, La Tronche, France; ^6^ Laboratory of Hematology and Tumor Bank, INSERM UMR-S 1172, Cancer Research Institute of Lille, CHRU of Lille, University Lille Nord de France, Lille, France; ^7^ CEA, Institut de Biologie François Jacob, SEPIA, Team Cellular Engineering and Human Syndromes, Université Paris-Saclay, Fontenay-aux-Roses, France; ^8^ Université Grenoble Alpes, Laboratory of Fundamental and Applied Bioenergetics, and Environmental and Systems Biology, Grenoble, France; ^9^ INSERM U1055, Grenoble, France; ^10^ CEA-Grenoble, Bioscience and Biotechnology Institute, Grenoble, France

**Keywords:** myelodysplastic syndromes, deferasirox, iron chelation, erythropoiesis, oxidative stress

## Abstract

Anemia is a frequent cytopenia in myelodysplastic syndromes (MDS) and most patients require red blood cell transfusion resulting in iron overload (IO). Deferasirox (DFX) has become the standard treatment of IO in MDS and it displays positive effects on erythropoiesis. In low risk MDS samples, mechanisms improving erythropoiesis after DFX treatment remain unclear. Herein, we addressed this question by using liquid cultures with iron overload of erythroid precursors treated with low dose of DFX (3μM), which corresponds to DFX 5 mg/kg/day, an unusual dose used for iron chelation. We highlight a decreased apoptosis rate and an increased proportion of cycling cells, both leading to higher proliferation rates. The iron chelation properties of low dose DFX failed to activate the Iron Regulatory Proteins and to support iron depletion, but low dose DFX dampers intracellular reactive oxygen species. Furthermore low concentrations of DFX activate the NF-κB pathway in erythroid precursors triggering anti-apoptotic and anti-inflammatory signals. Establishing stable gene silencing of the Thioredoxin (TRX) 1 genes, a NF-κB modulator, showed that fine-tuning of reactive oxygen species (ROS) levels regulates NF-κB. These results justify a clinical trial proposing low dose DFX in MDS patients refractory to erythropoiesis stimulating agents.

## INTRODUCTION

Myelodysplastic syndromes (MDS) are a rare group of heterogeneous clonal stem cell disorders characterized by dysplasia on myeloid cell lines leading to ineffective hematopoiesis and evolution to acute myeloid leukemia [1]. Low-risk MDS (low or intermediate-1 risk from international prognostic scoring system IPSS [2] and recently IPSSR [3] are characterized by increased intramedullary apoptosis of hematopoietic progenitors leading to inefficient hematopoiesis and cytopenia [4, 5].

Anemia is the most frequent cytopenia in MDS and most patients require red blood cell (RBC) transfusion resulting in the development of iron overload (IO). IO is a common complication of MDS management, decreasing life expectancy due to cardiac and liver failure [6]. As a consequence, most patients become transfusion-dependent and have to be treated with iron chelation therapy (ICT) such as deferoxamine, a classical iron-chelating agent which has to be administered intravenously. Deferasirox (DFX) with its high lipophilic property and oral administration has become a standard for IO treatment [7, 8]. The standard dose is ranging from 20 to 30 mg/kg/day. Many studies have shown the efficiency of DFX to decrease iron burden in lower risk MDS patients under red blood cell transfusion programs [9, 10]. Interestingly, ICT with DFX seems to have positive effects on hematopoiesis in some MDS patients leading to reduction of RBC transfusion or even transfusion independence [11–20].

In this study, we bring insight on an original effect of DFX in iron-overloaded condition. Strikingly, low dose DFX improves erythropoiesis *in vitro*, through reduction of ROS levels and activation of NF-κB. We demonstrate that ROS levels finely regulate the activation of NF-κB in the implemented cellular model.

## RESULTS

### Low dose of deferasirox has a beneficial effect on proliferation rates

A total of 27 low risk MDS samples were analyzed (more details are given in Table 1). Because of the varying initial proportion of CD34^+^ cells and the inherent heterogeneity of proliferation capacities for each myelodysplastic samples, proliferation rates were given as a proliferation ratio (PR) consisting in the number of cells counted at each day of analysis divided by the initial number of CD34^+^. In a set of preliminary experiments, we tested various DFX doses, ranging from 1, 3, 5, 7.5, 10 and 20μM onto hematopoietic stem progenitor cells (HSPCs) stemming from 3 MDS samples. It is noteworthy that 20μM corresponds to the plasma level of patients receiving 20 mg/kg/day of DFX for iron chelation use. Our results indicated that above 7.5μM DFX, there were no positive effects on cell proliferation in erythroid progenitors of MDS samples (data not shown). Consequently, all further experiments were carried out with 3μM DFX, corresponding to an oral dose of 5 mg/kg/day in patients, which will be called low dose (LD) DFX throughout the text.

**Table 1:** Samples characteristics

Patient	Date of birth	Diagnosis	Karyotype	IPSS-R
Patient 1	27/04/1926	RCMD	46, XY (20)	**low**
Patient 2	18/08/1934	RCMD	46, XY (20)	**low**
Patient 3	01/04/1941	CMML	45, X,-Y, del(13)(q12q21)[17]/45, sl, del(7)(q21q35)[3]	**high**
Patient 4	19/08/1938	RCMD	46, XX (20)	**low**
Patient 5	24/06/1957	RCMD	46, XY (20)	**low**
Patient 6	02/05/1951	RCMD	46, XY (20)	**low**
Patient 7	16/02/1947	ICUS	46, XX (20)	**low**
Patient 8	10/10/1955	RCMD	46, XX (20)	**low**
Patient 9	09/09/1932	RCMD	46, XX (20)	**low**
Patient 10	14/04/1959	RCMD	46, XX (20)	**low**
Patient 11	10/04/1953	RCMD	46, XY (20)	**low**
Patient 12	25/01/1928	RCMD	46, XY (20)	**intermediate**
Patient 13	28/02/1941	CMML	46, XY (20)	**low**
Patient 14	03/11/1947	RCMD	46, XY (20)	**low**
Patient 15	09/07/1936	RCMD	46, XX (20)	**low**
Patient 16	23/12/1968	RN	46, XY (20)	**low**
Patient 17	22/10/1939	RCMD	46, XX (20)	**low**
Patient 18	26/04/1933	RCMD	46, XY (20)	**low**
Patient 19	07/06/1940	RCMD	46, XY (20)	**low**
Patient 20	16/08/1924	ICUS	46, XX (20)	**low**
Patient 21	15/09/1935	RCMD	46, XX (20)	**low**
Patient 22	28/06/1925	CMML	46, XX (20)	**low**
Patient 23	15/11/1951	CMML	46, XX (20)	**low**
Patient 24	28/02/1941	CMML	46, XY (20)	**low**
Patient 25	31/07/1945	CMML	46, XY (20)	**low**
Patient 26	10/06/1973	RN	46, XY (20)	**low**
Patient 27	13/02/1935	CMML	46, XY, del(16)(q12q23)[10]/46, sl, del(9)(q13q33)[3]/46, sl,+del(16)(q12q23)[1]/	**high**

Diagnosis: CMML: chronic myelo-monocytic leukemia; ICUS: idiopathic cytopenia of undetermined significance; RCMD: refractory cytopenia with multilineage dysplasia; RN: refractory neutropenia.

**Figure 1:**
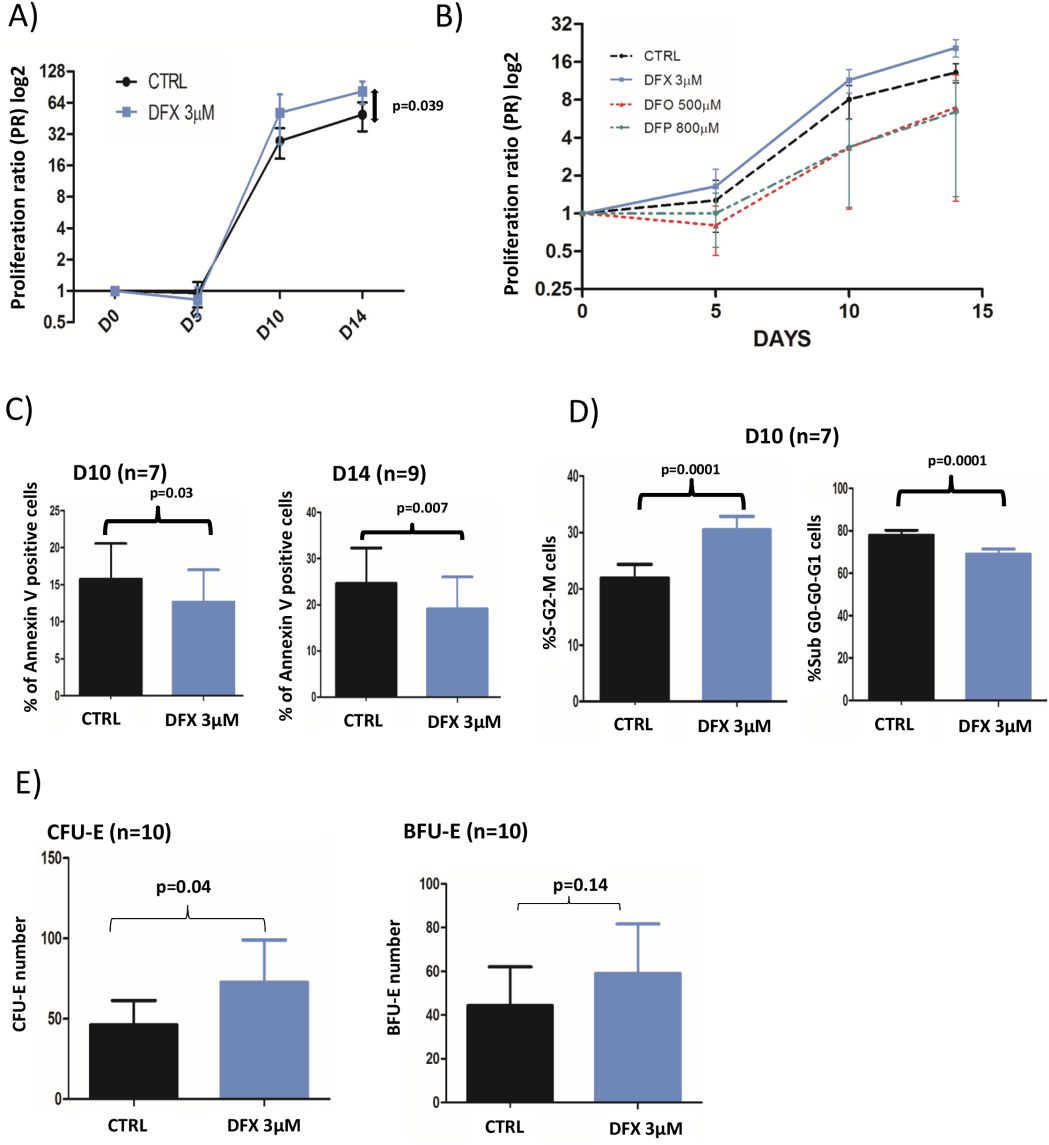
**(A)** Cell proliferation rates with or without 3 μM DFX (n=18). Proliferation rates were given as a proliferation ratio (PR) consisting in the number of cells counted at each day of analysis divided by the initial number of CD34^+^. CD34^+^ from myelodysplastic patients cells treated with DFX 3μM had a better proliferation rates (p=0.039) at the end of the cell culture period (day 14). **(B)** Proliferation rates after DFX (3μM) treatment in comparison with deferoxamine (DFO; 500μM) or deferiprone (DFP; 800μM) for 3 samples of CD34^+^ from myelodysplastic patients. **(C)** Results of apoptosis assay (% of annexin V positive cells) assessed by flow cytometry using annexin V staining. **(D)** Cell cycle was analyzed by flow cytometry with DAPI staining (n=7) and the two histograms correspond to cells in mitosis and S-phase (left) or in other phases of the cycle (right). **(E)** Clonogenic assays were started at D5 (n=9) of the erythroid differentiation model. BFU-E: Burst Forming Unit-Erythroid; CFU-E: Colony Forming-Unit-Erythroid.

For control condition (CTRL) and LD DFX respectively, PR were 27.6 (2.1-134.4) and 51.3 (3-440.7) (p=0.19) at D10 of erythroid differentiation, and 49.2 (3.2-207.9) and 82.2 (5.6-229.8) (p=0.039) at D14 (Figure 1A). These results showed an increased proliferation rate of erythroid progenitors derived from MDS samples 14 days after LD DFX treatment as compared to CTRL. Importantly, LD DFX did not affect the proliferation rate of HSPCs from healthy donors (n=5) which strongly suggests a specific role of LD DFX on MDS samples only (Supplementary Figure 1). We also sought to determine whether these proliferation-enhancing effects were specific to the chelating function of DFX. For that, we have tested two other iron chelators routinely used in clinical practice, desferoxamine (DFO) and deferiprone (DFP). In order to determine the concentrations of DFO and DFP which trigger similar iron chelating effects as LD DFX, we measured the total iron concentration in leukemia K562 cells by Inductively Coupled Plasma-Mass Spectrometry (ICP-MS) (data not shown). We then transposed those concentrations in the erythroid differentiation model, and impaired cell proliferation after either DFO or DFP treatment was observed as compared to the control conditions (Figure 1B), in contrast to the LD DFX effect. These expected results with DFO or DFP confirmed previously observed iron withdrawal effects on cell proliferation [21]. Altogether, these results demonstrate that LD DFX stimulates cell proliferation of MDS erythroid progenitors independently of iron removal.

### Increased proliferation rates with LD DFX are due to a larger proportion of cycling cells, less apoptosis but no effects on erythroid differentiation

LD DFX entailed fewer apoptotic cells as compared to control, at D10 (n=7), with 13.8% (0.7-35.9) *versus* 17.5% (1.1-39.8%) (p=0.03) respectively, and at D14 (n=9) with 19.1% (1.2-70) *versus* 24.7% (2.7-81) (p=0.007) respectively (Figure 1C). Besides, we also observed more cycling cells with LD DFX at D10 (n=7) with 30.5% of S-G2-M cells (19-34.5) for DFX *versus* 21.9% (11.8-29.1) for CTRL (p=0.0001) (Figure 1D). At D14, there was no more statistical difference (data not shown).

The clonogenic properties were probed on 10 different MDS samples (Figure 1E). We observed an increased number of CFU-E colonies with DFX *versus* CTRL with on average 72.7 (15-261) and 46.5 (3-174.5) colonies respectively (p=0.04). However, no significant differences were detected for BFU-E, between DFX and CTRL: 59 (23-192.5) *versus* 44.3 (3-179) (p=0.14). It was noteworthy that LD DFX did not accelerate the different steps of erythroid differentiation (Supplementary Figure 2).

### Induction of the NF-κB pathway by LD DFX

**Figure 2:**
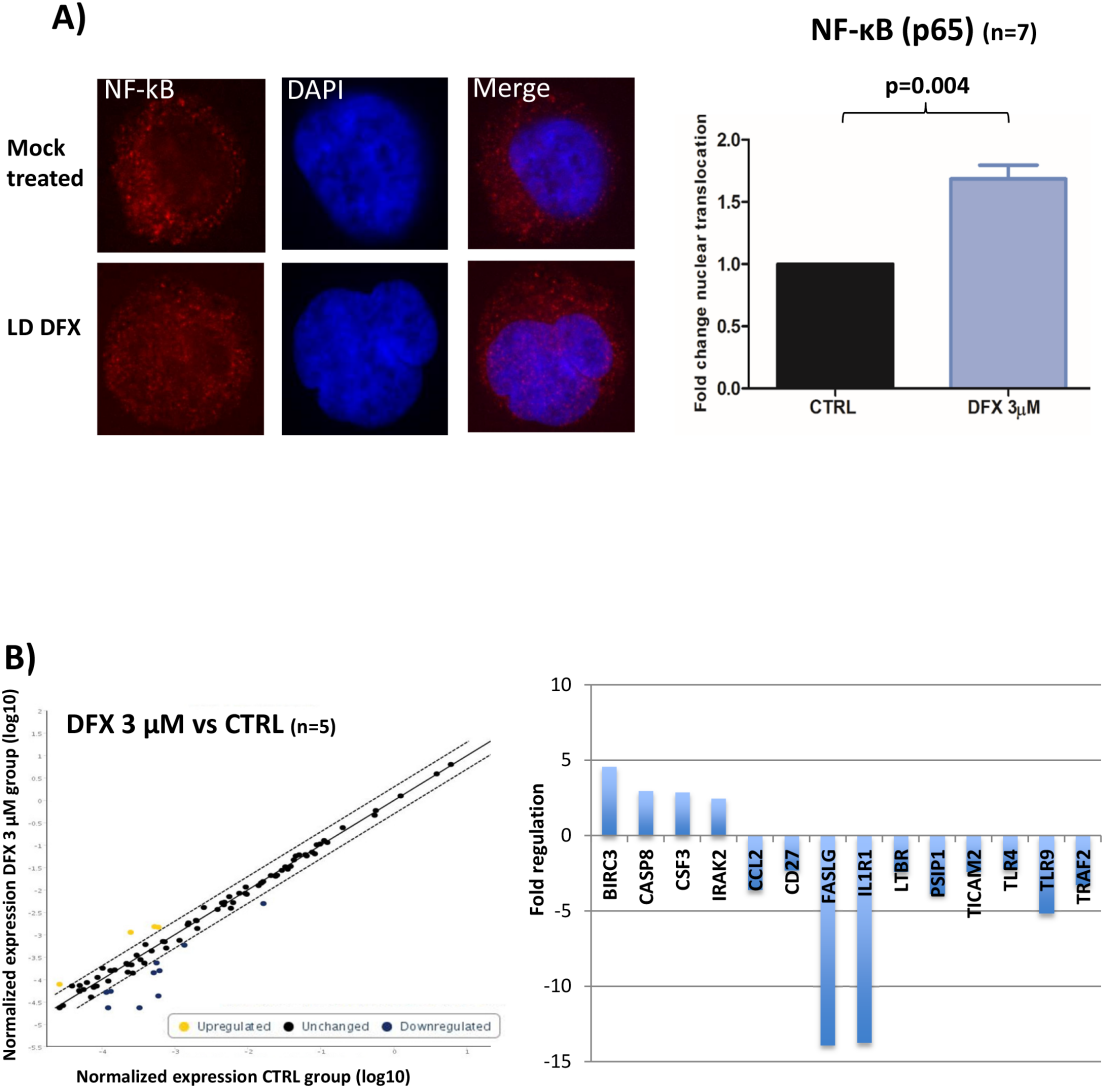
**(A)** Results of confocal immunofluorescence microscopy assays for NF-κB (n=7). Nuclei are stained in blue; the red signal represents the p65 subunit of NF-κB; x 63. Histogram representing the fold of change of NF-κB nuclear translocation. Data were analyzed by a specific software (ICY) to determine the nuclear fluorescence of NF-κB in the nucleus. Data were normalized to the CTRL condition in each case. **(B)** Results of the RT-qPCR microarray plates used to assess the impact of DFX on the expression of 84 known gene targets of NF-κB activation (n=5). Scatter plots represent the normalized expression of NF-κB targeted genes between DFX and CTRL conditions. The central line indicates unchanged gene expression. The dotted lines indicate the selected 2-fold regulation threshold. Data points beyond the dotted lines in the upper left area (yellow) are over expressed genes and those in the lower right sections (blue) are under expressed genes. Baculoviral IAP Repeat Containing 3 (BIRC3), caspase 8 (CASP8), Colony Stimulating Factor 3 (CSF3), and Interleukin 1 Receptor Associated Kinase 2 (IRAK2). Fas Ligand (FASLG), Interleukin 1 Receptor Type 1 (IL1R1), Toll Like Receptor 9 (TLR9), PC4 and SFRS1 Interacting Protein 1 (PSIP1), C-C Motif Chemokine Ligand 2 (CCL2), TNF Receptor Associated Factor 2 (TRAF2), Lymphotoxin Beta Receptor (LTBR), Toll Like Receptor Adaptor Molecule 2 (TICAM2), CD27, and Toll Like Receptor 4 (TLR4).

We then investigated which signaling pathways could be involved in the functional effects observed after DFX treatment. As evidenced by flow cytometry analysis, fluorescence intensities of either pAKT (Ser473), pS6 ribosomal protein (Ser235/236) or pERK (p44/42 MAPK) in 3 MDS samples treated with DFX (3μM) were not enhanced as compared to mock treated cells (data not shown). We then probed two major transcription factors required for hematopoiesis and tumorigenesis: NF-κB (nuclear factor-kappa B) and FOXO3a (a member of the Forkhead box O). We failed to detect any change of location of FOXO3a by confocal microscopy as compared to mock treated cells (data not shown) (n=3). Regarding NF-κB nuclear recruitment after LD DFX treatment, we observed an increased NF-κB nuclear translocation (n=7) with an average fold of 1.69 (1.21-2.05) (p=0.004), which could correspond to an enhanced activation of the NF-κB pathway (Figure 2A).

We then analyzed 84 target genes belonging to the NF-κB pathway by RT-qPCR microarray plates on five samples (D14) treated or not with LD DFX. Four genes were over-expressed with LD DFX with a fold change > 2 and ten genes were down regulated (Figure 2B). Collectively, these results suggest enhanced anti-apoptotic (BIRC3, CSF3 and FASLG) and anti-inflammatory responses (TLR9, TLR4, LTBR, and TRAF2) as a result of the downstream NF-κB activation.

Moreover, we attempted to determine which of clonal or normal hematopoiesis could be amplified by LD DFX. To identify the molecular pattern of cells after DFX treatment, we applied the erythroid differentiation model to 2 patients, one harboring mutation in the IDH1 (Isocitrate Dehydrogenase 1) gene, and the second in the MPL (MPL proto-oncogene, thrombopoietin receptor) and the SRSF2 (Serine and arginine Rich Splicing Factor 2) genes. The evolution of the variant allele frequency (VAF) was followed by Next Generation Sequencing (NGS) after different times of treatment (Supplementary Figure 3). Interestingly, we saw a slight decrease of the VAF at the end of the culture period for cells treated with DFX strengthening the notion that DFX could maintain a normal hematopoiesis.

### DFX functional effects are independent of functional iron depletion

**Figure 3:**
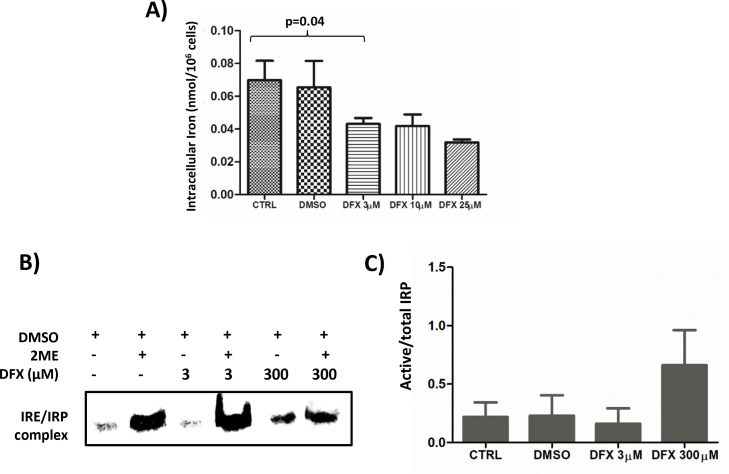
**(A)** Decreased intracellular iron content after DFX treatment as measured by ICP-MS. K562 cells were treated with increased doses of DFX for 48 hours (n=4). **(B)** Representative (n=2) Iron Regulatory protein (IRP) activity in K562 cells treated with increasing concentrations of DFX. IRP activity was assessed by REMSA using an Iron Responsive Element (IRE) biotin-labeled probe. The apo-IRP form was activated after treatment with 2-mercaptoethanol (2ME). **(C)** Histogram representing the ratio of the measured IRP activity divided by the total of IRP activity revealed by the 2-mercaptoethanol treatment (n=2).

Since the functional effects of DFX are usually assigned to its iron chelating properties, intracellular iron concentrations were measured by Inductively Coupled Plasma Mass Spectrometry (ICP-MS) after treatment of K562 cells with increasing doses of DFX for 48 hours (n=4). There was a dose-dependent decrease of intracellular iron with DFX reaching statistical significance (p=0.04) starting from 3 μM and above (Figure 3A).

Cellular iron homeostasis is regulated by iron regulatory proteins (IRP) which are cytoplasmic RNA-binding proteins either stabilizing mRNAs by recognizing RNA motifs, termed Iron-Responsive Elements (IRE), located on 3′ untranslated regions (3′UTR), such as for transferrin receptor 1, or impairing translation of those for which IRE are on the 5′UTR, as for the ferritin subunits [22]. To determine if LD DFX perturbs iron homeostasis, the activity of the Iron Regulatory Proteins (IRP) was measured by RNA electrophoretic mobility shift assay (REMSA) on K562 cells treated with increasing doses of DFX (Figure 3B). The cell line was grown in iron-overloaded medium to mimic the conditions experienced by MDS progenitors. An increase of the IRP activity present in K562 cells was only seen by applying 300 μM of DFX to the culture for 48 hours (Figure 3B lane 5), whereas 3 μM DFX (lane 3) did not change the activity present in control cells (lane 1). In all cases, this basal activity reflected only a minority (< 10% Figure 3C) of the total IRP present in the samples, as revealed by treatment of the lysates with β-mercaptoethanol (lanes 2,4,6). These results indicate that, although LD DFX induced a decrease of total cellular iron (Figure 3A), this low dose did not change the availability of functional iron as detected by the activity of the IRP.

### LD DFX protects cells from oxidative stress

**Figure 4:**
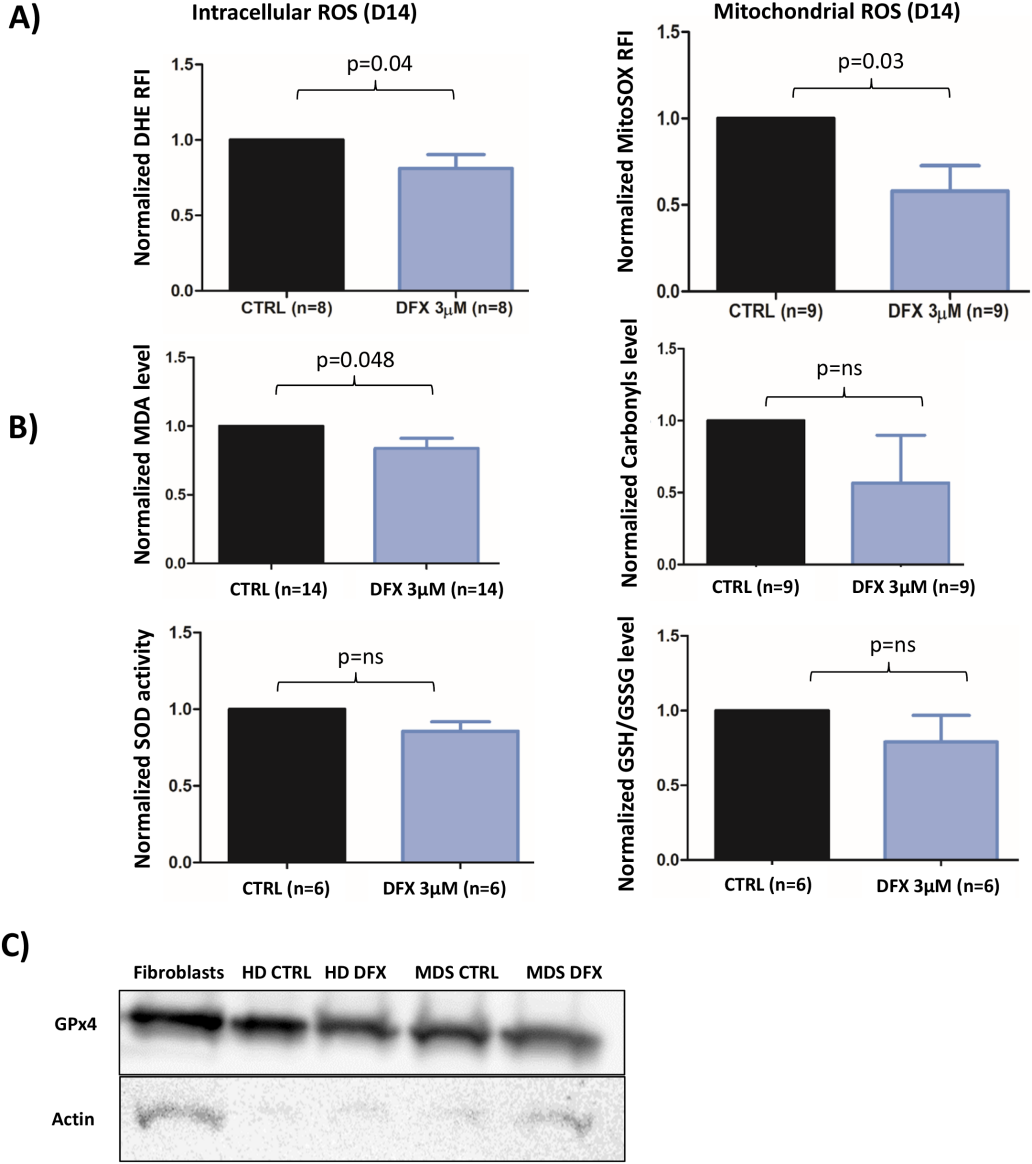
**(A)** Flow cytometry analysis of intracellular ROS with DHE staining (n=8) and mitochondrial ROS with MitoSOX probe (n=9) at D14 of the cell culture procedure. The data are normalized to the CTRL condition in each case. **(B)** Levels of MDA, Carbonyls, Glutathione and SOD (SOD 1-3) activity normalized to the CTRL condition. **(C)** Western blot of GPX4 for MDS patient (three samples were pooled) and healthy donor (n=1). Fibroblasts were used as a positive control.CTRL: control; DMSO: Dimethylsulfoxyde; REMSA: RNA electrophoretic mobility shift assay; HD: healthy donor; IRP: Iron response protein; IRE: Iron Responsive Element; MDA: Malondialdehyde; ROS: reactive oxygen species; SOD: superoxide dismutase.

We assessed ROS levels in 9 different MDS samples during erythroid differentiation after 10 (D10) or 14 (D14) days in culture (Figure 4A). Intracellular oxidizing species, such as the superoxide radical anion, were measured with the fluorescent DHE probe. At D14, LD DFX (n=8) decreased the level of DHE-reactive species (average RFI: 6.86 (3.52-10.12)) as compared to CTRL (average RFI: 9.05 (4.77-15.76)) corresponding to a 20% average decrease (p=0.04). In the same experimental conditions, the mitochondria-targeted superoxide-reacting MitoSOX probe detected an average decrease of 58% (p=0.03) with LD DFX (n=9) (average RFI: 21 (7.33-33.2) compared to CTRL (average RFI: 30.7 (13.7-48.3)).

Thereafter, we sought to determine whether some metabolites and antioxidant enzymes participating to the cellular redox homeostasis could be regulated by LD DFX. The Malondialdehyde (MDA) level (p<0.05) was slightly decreased with LD DFX, but no impact of DFX on carbonyls levels was observed (Figure 4B). Neither SOD (SOD1-3) activity nor the GSH/GSSH ratio were significantly changed after LD DFX treatment (Figure 4B).

Remarkably, GPx activity was not detected in 6 different MDS samples in the iron overloaded system and, likewise, in 4 different MDS samples cultured in the more conventional, non-iron overloaded medium (data not shown). GPx4 and GPx8 were recently involved in redox homeostasis during erythroid differentiation [23]. They were tested by RT-qPCR in seven different MDS samples cultured in iron-overloaded medium. GPx8 mRNA was only present in 1 sample, whereas GPx4 mRNA was present in all MDS samples without difference in relative expression in the presence of LD DFX (data not shown). Western blots confirmed the presence of the GPx4 protein in samples of MDS patients treated or not with LD DFX and also in a healthy donor sample (Figure 4C). We performed lysis of 3 MDS samples under nitrogen to avoid oxidative damage to the highly reactive active site selenocysteine of GPx4. As expected, GPx activity was detected, meaning that this activity was inhibited under oxygen-saturated conditions in the conventional lysis procedure. GPx activity was not modified by the LD DFX treatment (data not shown).

These results outlined that the redox molecular shift associated with decreased oxidizing species induced by LD DFX was responsible for the lowered MDA values (Figure 4B). Interestingly, lipid peroxidation is a hallmark of iron-dependent regulated necrosis, also termed as ferroptosis, which might be indicated by lowered MDA values. Therefore, we asked whether LD DFX might protect cells from ferroptosis when iron overload conditions were applied. For this purpose, the gene expression levels of 6 biomarkers of ferroptosis [24] were measured by RT-qPCR. After LD DFX treatment, the expression of these genes remained unchanged as compared to CTRL (Supplementary Figure 4). In addition, DFX did not interfere with the GPx4 activity, another hallmark of ferroptosis. Therefore, ferroptosis is not a death mechanism impacted by LD DFX under our experimental conditions.

### NFKB is regulated by an extremely fine control of ROS levels

**Figure 5:**
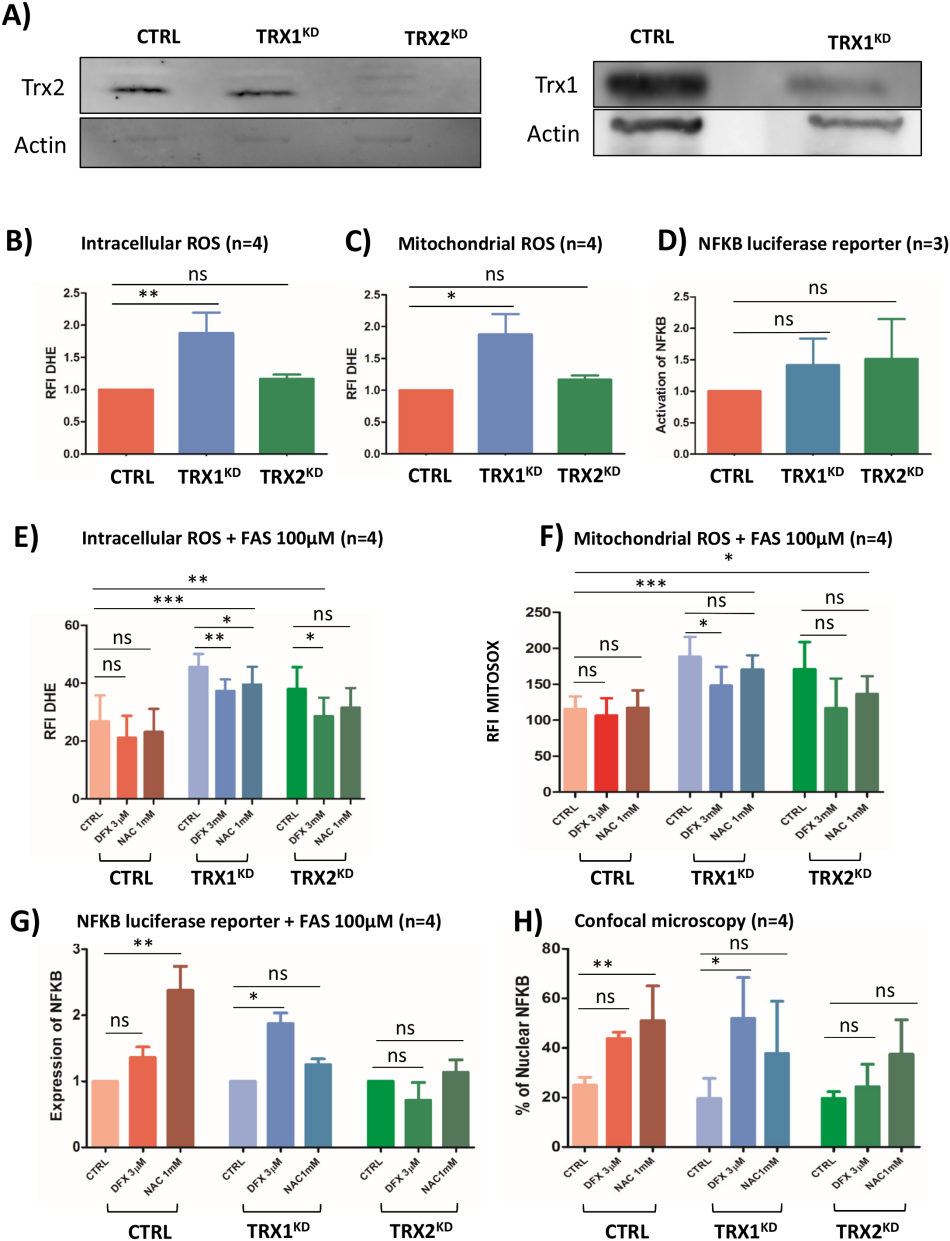
**(A)** Western blot analysis of Trx1 and Trx2 protein levels in stable TRX1^KD^ and TRX2^KD^ K562 cells. Trx1 and Trx2: 13 kDa; actin: 42 kDa. **(B)** Flow cytometry analysisof intracellular ROS with DHE staining (n=4) in CTRL and stable TRX^KD^ K562 cells without iron overload. **(C)** Same experiment as in B for mitochondrial ROS with MitoSOX probe (n=4). **(D)** Assessment of NF-κB activity with a luciferase reporter assay (n=4) in TRX^KD^ K562 cells without iron overloaded condition. **(E)** Same experiment as in B with FAS (100μM) and DFX 3μM or NAC 1mM. **(F)** Same experiment as in C with FAS (100μM) and DFX 3μM or NAC 1mM. **(G)** Same experiment as in D with FAS (100μM) and DFX 3μM or NAC 1mM. **(H)** Quantification of NF-κB translocation by confocal microscopy for K562 cells as described in **(G)**. (FAS: ammonium sulfate to the medium, DFX: deferasirox, NAC: N-acetylcysteine; RFI: ratio of fluorescence; KD: knock-down; TRX: thioredoxin; ^*^: <0.05; ^**^: <0.001; ^***^: <0.0001).

To demonstrate the tight correlation between DFX, ROS downregulation and activation of the NFκB pathway, we have engineered a cellular model by a strategy described previously [25] to silence thioredoxin (TRX) gene expression by stable RNA interference. The two isoforms of thioredoxin, Trx1 expressed in the cytoplasm and nucleus and Trx2 localized within the mitochondria, contribute to the intracellular redox equilibrium. Trx play a dual role by inhibiting NF-kB nuclear translocation under oxidizing conditions, and by enhancing its binding to DNA [26]. TRX^KD^ knock-down cells were analyzed by Western blot (Figure 5A). As a CTRL, we established K652 cells stably expressing an inefficient shRNA sequence which has been widely used in previous studies [27].

Without adding excess iron in the growth medium, TRX1^KD^ cells exhibited a 1,8-fold increase of both DHE (p=0.008) and MitoSOX fluorescence (p=0.01) (Figure 5B and 5C). Conversely, in this condition, TRX2^KD^ cells failed to exhibit significant changes. Hence, Trx1 might be a more efficient factor against the oxidative stress detected by these probes than Trx2. Under the same conditions, NF-κB was not activated in both TRX1^KD^ and TRX2^KD^ cells (Figure 5D) as assessed by a luciferase reporter assay. The cellular redox equilibrium was challenged by adding ferric ammonium sulfate (FAS, 100μM), LD DFX, or a conventional antioxidant molecule, N-acetylcysteine (NAC 1mM) to the culture medium of K562 cells. In CTRL K562 cells, addition of FAS induced a 1.8-fold increase of DHE fluorescence (Figure 5E) and a 8-fold increase of MitoSOX signal (Figure 5F) in comparison to basal conditions (Figure 5B and 5C).

When excess iron (FAS) was added, the fluorescence of TRX1^KD^ K562 cells exhibited a 3.1-fold increase and 13-fold increase for DHE and MitoSOX signals, respectively, as compared to basal conditions (Figure 5E and 5F *versus* 5B and 5C, blue columns). Interestingly, LD DFX induced a significant decrease of fluorescence (20%) for both DHE (p=0.001) and MitoSOX (p=0.02), whereas NAC only decreased DHE fluorescence (p=0.04). Furthermore, DFX triggered NF-κB activation (p=0.03) in TRX1^KD^ K562 cells in the presence FAS (100 μM) (Figure 5G, blue columns), which was consistent with the increased nuclear translocation of NF-κB visualized by immunofluorescence microscopy (p=0.04) (Figure 5H, blue columns). Whereas NAC induced a minor decrease of ROS level compared to DFX (Figure 5E), it was not associated with an increase of NF-κB activation measured by both confocal microscopy and luciferase reporter.

In TRX2^KD^ K562 cells, we observed a 2.6 and 11.8-fold increase for DHE and MitoSOX signals, respectively, as compared to basal conditions (Figure 5E and 5F *versus* 5B and 5C, green columns). NAC was not associated with any significant decrease of ROS levels. In contrast, LD DFX significantly decreased the level measured with the DHE probe, although this decrease was not significant with MitoSOX. However, DFX and NAC did not induced NF-κB activation in TRX2^KD^ K562 cells (Figure 5G and 5H, green columns).

Collectively, these results indicated that NF-κB activation in our iron overload model depended on Trx1. NF-κB is finely regulated by the levels of oxidative species and it is consistently increased by LD DFX under these conditions.

### Translational application of low dose of DFX

**Figure 6:**
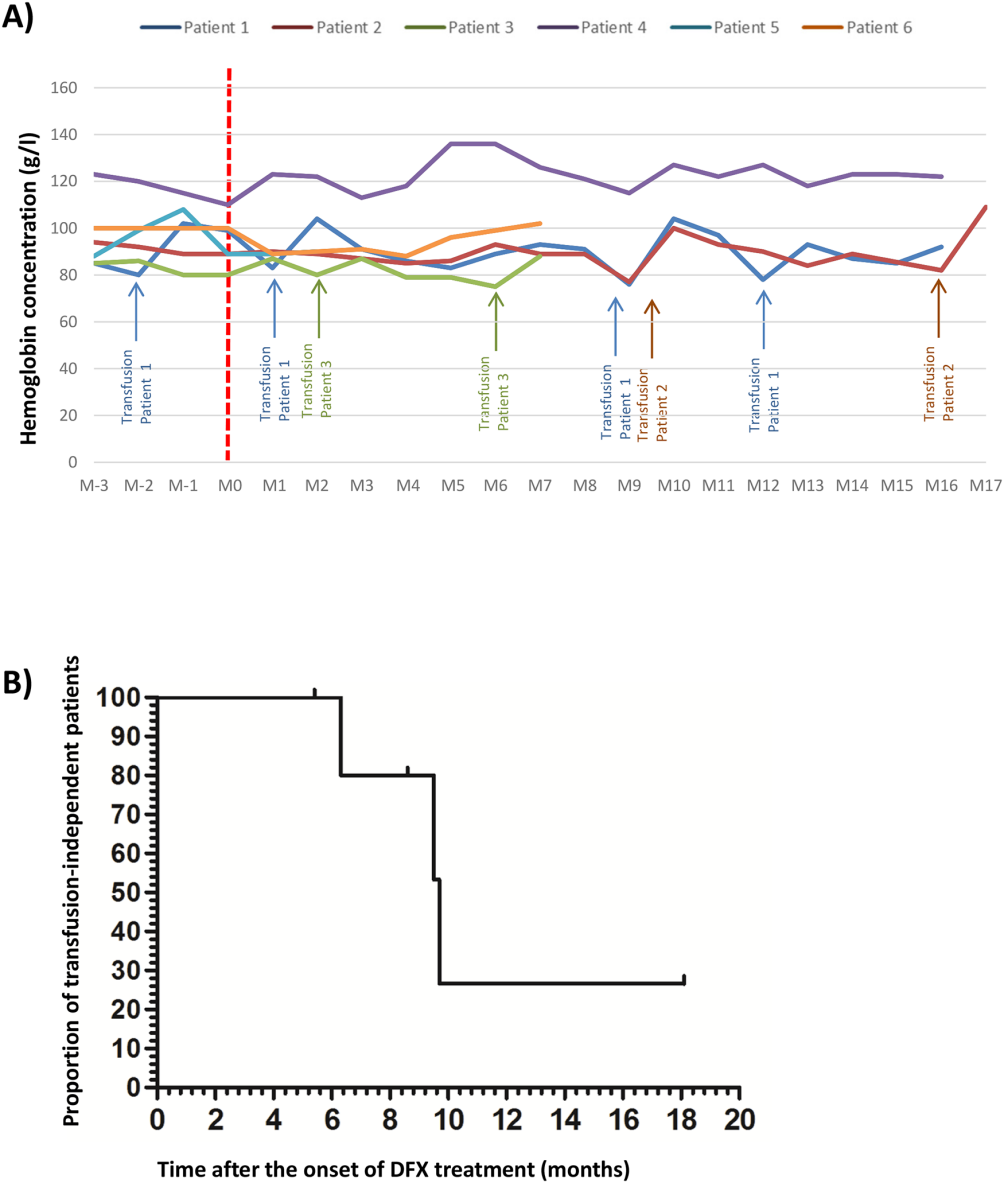
**(A)** Evolution of the hemoglobin level for 6 anemic patients with low risk MDS and refractory to erythropoiesis stimulating agents treated by low dose of deferasirox (DFX) before and after the beginning of DFX; initial hemoglobin level = T0 DFX). **(B)** Evolution of the proportion of untransfused patients after DFX treatment for the 6 anemic patients with low risk MDS and refractory to erythropoiesis stimulating agents treated by low dose of DFX.D: day; Hb: hemoglobin level, M: month.

Based on these *in vitro* results, and because deferasirox is currently used treatment of iron overload, its effectiveness at low dose (5 mg/kg/day corresponding to a plasmatic level of 3μM) in MDS patients was evaluated locally in our clinical center. At the usual recommended dosage (20 mg/kg/day), DFX usually induces many side-effects and poor tolerance. Six anemic patients (2 Refractory cytopenia with multilineage dysplasia (RCMD), 1 idiopathic cytopenia of undetermined significance (ICUS), 1 refractory anemia with ringed sideroblasts (RARS), 1 refractory anemia (RA) and one MDS with del5q) were followed and treated with 5 mg/kg/day of DFX. These patients displayed the following inclusion criteria: low risk MDS, anemia refractory to erythropoiesis stimulating agents or high endogenous level of EPO (>500IU/L) and plasma ferritin (< 1000μg/l). They were treated prospectively with a low dose of DFX (5 mg/kg per day) corresponding approximately to the 3μM concentration used in the *in vitro* analyses according to blood plasma (6.31μM [1.072-16]) and medullar plasma (2μM [1.072-2.546]) level measurements. The plasma level of deferasirox was checked *ex vivo* and corrections of its posology were made accordingly. Three of the patients included required monthly RBC transfusions before the beginning of the DFX treatment. The hemoglobin levels of the patients did not reach normal recommended values, but they were stabilized leading to transfusion independence or diminution of the transfusion frequency (Figure 6A). Currently, after 2 years of follow-up, the median time to transfusion independence after DFX treatment was 9.6 months (Figure 6B). Moreover, if we consider the definition of transfusion independence according to IWG 2006 [28] criteria, all patients treated with LD DFX became transfusion-independent. No side-effects were reported.

## DISCUSSION

In this study, we have demonstrated that low doses of deferasirox below 7.5 μM had a beneficial effect on erythropoiesis in low risk MDS patients *in vitro*. The higher proliferation rate observed with LD DFX is due to the combination of increased cycling cells and protection against apoptosis linked to enhanced NF-κB activation regulated by ROS levels.

The aim of our study was to unravel the positive effect of LD DFX on erythroid proliferation. Indeed, we have tested elevated concentrations of DFX, such as 20μM, which is currently measured in the plasma of patients taking DFX for iron chelation purpose (between 20 to 30 mg/kg/day). However these high doses were toxic for erythroid progenitors in our cellular model. Pullarkat et al [29] have demonstrated a benefit of low dose of deferasirox (5μM) on proliferation and toxicity for hematopoietic progenitors at higher doses, which was confirmed herein. Moreover, Messa et al [30] observed that a higher dose of DFX (50μM) induced apoptosis in two leukemic cell lines. Paubelle et al [31] had successfully used high doses of DFX (30 mg/kg) for treating elderly leukemic patients attesting the antileukemic effects of these doses. Altogether, these controversial results stressed that DFX displays a biphasic mode of action, with erythroid proliferation of progenitors of MDS patients at low doses and inhibition at the highest doses. For better understanding of the consequences of a LD DFX treatment on hematopoiesis, we have followed the clonal expansion of a mutated clone in the implemented liquid culture model. Regarding the results of the NGS experiments, we could rule out this possibility. LD DFX seemed unable to promote clonal hematopoiesis despite NF-κB activation which has been proved to be activated in high risk MDS [30]. Therefore, LD DFX maintained a normal hematopoiesis expansion.

One of the main aspects of our work was to investigate more deeply the connections between exposure to LD DFX and iron homeostasis by measuring the Iron Regulatory Proteins (IRP) activity: the lower the functionally available iron, the higher the IRP activity [21]. Recently, Pourcelot et al [21] have introduced the notion of functional intracellular iron concentration (FIC). FIC is the needed iron for cell homeostasis and IRP activity is the better way to measure it. In contrast, labile iron concentration (LIC) is the redox fraction implicated in Fenton reaction. In this study, our results indicate that LD DFX induced a decrease of total cellular iron leading to ROS protection probably due to a decrease of LIC but this decrease did not change the availability of FIC detected by the activity of the IRP. Herein, we demonstrate that LD DFX functional effects on iron loaded hematopoietic progenitors are not due mainly to the iron chelation properties of the drug but essentially to the protective effect of LD DFX on oxidative stress. Reactive oxygen species play an important role in the physiopathogenesis of MDS [32] and in other myeloid malignancies [33]. In our study, we showed that MDS progenitors, in the presence of iron excess, produce high levels of cytoplasmic and mitochondrial ROS. Malondialdehyde (MDA), the most mutagenic product of lipid peroxidation of polyunsaturated fatty acids, also interacts with and modulates proteins. We highlighted that LD DFX decreased MDA levels. Lipid peroxidation, associated with decreased GPx4 activity, is a hallmark of ferroptosis which is a form of non-apoptotic programmed cell death induced by iron excess and dependent on GSH depletion [34]. We did not observe any modification in genes specifically involved in ferroptosis after DFX treatment, suggesting that LD DFX might protect cells from death by inhibiting apoptosis but not ferroptosis. Besides, we suggest that the protecting effects of DFX on lipid peroxidation is tightly correlated with ROS downregulation.

The main signaling pathways activated in erythroid progenitors are PI3K/AKT, mTOR, MAPkinase and NF-κB [35, 36]. In our cell model, we observed enhanced NF-κB nuclear translocation followed by activation of specific target genes strengthening that NF-κB was directly activated by LD DFX. Interestingly, we detected the repression of genes required during apoptosis and others involved in the inflammatory response of NF-κB mediated by IL-1 and TLR stimuli. In the context of increased inflammatory signals triggered by the S100A9 protein via TLR4 and the inflammasome NLRP3 [37], it was noteworthy that LD DFX inhibited both IL1R1 and TLR4 expressions.

NF-κB is one of the first transcription factor that was recognized to be redox-regulated. While some NF-κB-regulated genes are key factors in redox homeostasis, ROS can act as secondary messengers and interfere with NF-κB signaling pathways. Among the diversity of mechanisms controlling NF-κB activities, a major one depends on IκB inhibitors and IκB kinases (IKKs) for governing NF-κB activation, degradation, DNA binding and transcriptional activity [38]. While oxidative stress generally activates NF-κB [39], recent studies suggested that ROS could repress NF-κB pathway. Indeed, ROS appeared to activate NF-κB DNA binding activities during the early phase of oxidative stress, mainly by abrogating the inhibitory action of IκB inhibitors [40]. In later phases of oxidative injury, ROS may repress NF-κB. One possible mechanism is oxidation of IKKs, blocking IκK phosphorylation, and then hampering NF-κB activation [41]. In our model, it appears that decreased ROS levels induced by LD DFX can restore NF-κB activity through such a mechanism.

Our results supported that NF-κB signaling was modulated by a fine-tuning of the intracellular redox state. Because thioredoxins (Trx) are among the major endogenous redox-regulating molecules with thiol reducing activity, we have engineered stable K562 cells silenced for TRX1 or TRX2 genes [40]. The literature reveals controversial data concerning thioredoxin and NF-κB signaling. Trx could play dual and opposite roles toward NF-κB activities depending on its subcellular localization (cytoplasmic *versus* nuclear compartments). Trx1 could trigger NF-κB nuclear translocation and activation through a tight interaction with p50/RelA sub-units, protecting critical cysteines from oxidative injuries [42, 43]. Older studies also pointed out the negative and positive regulation of NF-κB by Trx [43–45]. In our cell model, we combined TRX1 and TRX2 knock-down with FAS culture conditions to modulate total cellular and mitochondrial ROS. We showed that DFX reduced the ROS amount and induced NF-κB activation in a Trx1-dependent manner. NAC induced decrease of ROS levels in TRX(1-2)^−/−^ conditions, but this decrease was not as important as that induced by low dose DFX. We can hypothesize that ROS levels need to be sufficiently decreased to induce NFKB activation.

The translational application in our center of low dose of DFX for 6 low risk MDS patients showed improvement of erythropoiesis and a longer median time to transfusion dependence: 9.6 versus 6.1 months, if we consider the results obtained in the European cohort concerning MDS EPO refractory patients [46]. These results provide a rationale for a French prospective clinical trial which will propose LD DFX (5 mg/kg/day) in MDS patients refractory to erythropoiesis stimulating agents (N° EudraCT: 2017-001258-33).

To summarize, we have shown novel effects of LD deferasirox on NF-κB by fine modulation of ROS levels in low risk MDS erythroid progenitors. These results suggest that protection of MDS patients’ bone marrow from oxidative stress should be considered early in the course of their disease, and before the development of a significant iron overload which requires high dose of DFX which does not result in erythroid improvement.

## MATERIALS AND METHODS

### Cell samples

Bone marrow from low risk myelodysplastic patients and healthy donors were collected by bone marrow aspiration after signed consent and approval by our local ethical committee. Bone marrow aspirations were done for MDS diagnosis and also for the exploration of all kinds of cytopenia. When bone marrow morphology was normal, it was considered as healthy donor. CD34^+^ selection is described in supplementary methods. The K562 chronic myelogenous leukemia cell line was originally obtained from the ATCC biological resource. The K562 cells were grown in RPMI medium, supplemented with 10% fetal bovine serum, 1% L-glutamine, 100 UI of penicillin/ml and 0.1 mg streptomycin/ml at 37°C with 5% CO2.

### Cell culture procedure for erythropoiesis study

We used a 2 step-liquid culture procedure as already described [47, 48]. Details are given in supplementary methods.

### Other methods

Description for all other assays and techniques are given in supplementary methods.

### Statistical methods

The significance of the experimental results was determined with a 2-tailed paired Student test. p≤0.05 was considered statistically significant. All statistical analyses were done with GraphPad Prism 5 software (http://www.graphpad.com/scientific-software/prism/). Data are mean with SEM.

## SUPPLEMENTARY MATERIALS FIGURES


