# Supplementary material for: Reactive oxygen species levels control NF-κB activation by low dose deferasirox in erythroid progenitors of low risk myelodysplastic syndromes
Source: Oncotarget. 2017 Nov 6;8(62):105510–24. doi: 10.18632/oncotarget.22299 (PMC5739655; doi:10.18632/oncotarget.22299)
Supplement: Supplementary file 1 [file oncotarget-08-105510-s001.pdf]

Reactive oxygen species levels control NF-κB activation by low dose deferasirox in erythroid progenitors of low risk myelodysplastic syndromes

SUPPLEMENTARY MATERIALS

CD34+ selection from primary samples

By density gradient using Ficoll (Eurobio), the mononuclear cells were isolated from total bone marrow. HSPCs (hematopoietic stem progenitor cells) were positively selected by paramagnetic iron-dextran particles directly conjugated to anti-CD34 monoclonal antibodies (CD34 MicroBead Kit, human, Miltenyi Biotec) by using an automatized system (MACS pro, Miltenyi Biotec). The HSPCs were counted using Malassez cells (Fastread, Biosigma) and their viability was assessed by trypan blue exclusion (Sigma Aldrich). The purity of all samples was checked by flow cytometry (BD FACS Canto II, BD Biosciences) with a specific CD34 fluorochrome-conjugated antibody (CD34APC, Miltenyi Biotec) and was superior to 90% for all samples used.

Erythroid liquid culture procedure

HSPCs were cultured in DMEM medium (Dulbecco's modified eagle Medium, Life technologies) containing 12% FCS (Life technologies), 1% BSA (Sigma Aldrich), 0.5 IU/mL rHu Epo (Retacrit™, Hospira), 50 ng/mL SCF (PeproTech), 40 ng/mL rHu IGF-1 (RD system), 1.10^{-6} mol/L dexamethasone (Sigma Aldrich) and 100µM of ammonium Fe^{3+} sulfate dodecahydrate (Sigma-Aldrich). They were incubated in 12-well plates at 37°C in a 5% carbon dioxide atmosphere for 10 days until they reached the basophilic erythroblast stage. At D10, the cells were placed in DMEM medium containing 15% FCS, 1% BSA with 300 mg/mL holotransferrin, 1 IU/mL rHu Epo, 1 IU/mL insulin (Sigma-Aldrich) and 100µM of Ammonium Fe^{3+} sulfate dodecahydrate for terminal erythroid differentiation until D14. Deferasirox (DFX) was supplied by Novartis. DFX was solubilized in pure dimethyl sulfoxide (DMSO, Sigma-Aldrich). Three different conditions were tested: control (CTRL), DFX and DMSO as vehicle for DFX. DFX and DMSO were added at day 0 and day 10 of the cell culture procedure.

Functional assays

Cell proliferation was assessed by cell counting using Malassez cells. Apoptotic cells were determined by the percentage of Annexin V positive cells (FITC Annexin V, BD Biosciences) by flow cytometry, as per the manufacturer's intructions. Cell cycle was studied by DAPI staining (4',6-diamidino-2'-phylindole, dihydrochloride, 1mg/ml, Thermo SCIENTIFIC). Briefly, cells were permeabilized with commercial solution (Cytofix/cytoperm™, BD Biosciences). After washes with Permwash™ (BD Biosciences), cells were incubated with DAPI at room temperature for 1 hour. DNA content was evaluated by flow cytometry. All flow cytometry analyses were done on BD FACS Canto II.

Erythroid differentiation: flow cytometry and cytospins

Cells were collected at D5, D10 and D14 and stained with fluorochrome-conjugated antibodies for 30 minutes at room temperature. To analyze global erythroid differentiation, transferrin receptor (CD71-APC-AlexaFluor® 750, IOTest, Beckman Coulter) and glycophorin A (Glycophorin A-VioBlue, clone: REA175, Miltenyi Biotec) were used. Three populations are described: proerythroblasts $CD71^+/CD235^-$, basophilic erythroblasts $CD71^+/CD235^+$ and orthochromatic erythroblasts $CD71^-/CD235^+$. For cytospin preparation, $2x10^5$ cells in 200µL of PBS BSA 1% were put at D10 on coated slides, using the Thermo Scientific Cystopin 4. The slides were stained with May-Grunwald-Giemsa (MGG) by an automatized way (Aerospray®Pro, Elitech).

Assessment of intracellular ROS by flow cytometry

We measured intracellular ROS level using dihydroethidium (DHE, InvitrogenTM) and mitochondrial ROS level using a specific probe MitoSOX ([3,8-phenanthridinediamine, 5-(60-triphenylphosphoniumhexyl)-5,6 dihydro-6-phenyl]; Molecular Probes, Invitrogen). The results are given by ratio of fluorescence RFI.

Intracellular pathway: immunofluorescence microscopy assays and flow cytometry

For confocal microscopy, at D10, 1.10^5 cells permeabilized following the same protocol as for cell cycle. Cytospins were prepared, saturated with PBS BSA 2% during 45 min. For FOXO3a staining, incubation was done overnight at 4°C and with a dilution of 1/400 concentration for primary antibody (anti-human FOXO3A,

clone 3F12, Sigma Aldrich). After a wash with PBS, the secondary antibody (Alexa-fluor 647 donkey anti-mouse, Life technologies, Eugene, USA) was incubated during one hour at room temperature at dilution of 1/200. After an ultimate wash with PBS, 5µL of a mix of Vectashield ™ (Vector laboratories) with Dapi 1/2000 (DAPI antifade ES, Cytocell) was put on cells. Preparations were read on spinning disk confocal microscope (IMIC 2.0 Till Photonics, FEI, Munich) with a camera (iXon 897, EMCCD, Andor). Then data were analyzed with Icy software (http://icy.bioimageanalysis.org). The same procedure was done for NF-κB (p*NF-κB* p65, clone L8F6, Cell Signaling) with a dilution of 1/400 for primary antibody and 1/200 for the secondary antibody.

For flow cytometry, at D10, cells were permeabilized and stained with pAKT (pAKT, alexa 488, Cell Signaling), ERK (pErk 1/2, Alexa 647, Cell Signaling), pS6 ribosomal protein (pS6, Alexa 647, Cell Signaling) during overnight at 4°C and with a dilution of 1/50 and then analyzed by flow cytometry.

Clonogenic assays

At D5, cells were harvested and were cultured in 12-well plates with 500µL of MethoCultTM H4434 Classic (Stemcell Technologies) at concentration of $1x10^4$ cells per well. All conditions were done in duplicate. Colony forming unit erythroid (CFU-E) and the burst forming unit erythroid (BFU-E) were counted after 7 days and 14 days of culture respectively based on morphological classical criteria. An external technician to the team read the colonies in "double blind" condition.

NF-κB pathway-specific expression array

The human NF-κB signaling targets RT^2 Profiler PCR array kit (SABiosciences) was used to assess the impact of DFX on the expression of 84 genes target of NF-κB according to the manufacturer's instructions.

Intracellular iron measurements

Three millions of K562 cells were incubated for 48h with increasing doses of DFX (from 3 to 200µM) in the same medium as described above plus 100µM of ammonium Fe^{3+} citrate. Cells were lysed by heat shock in water and intracellular iron concentrations were determined by Inductively Coupled Plasma-Mass Spectrometry (ICP-MS) using a XSERIES 2 analyzer (Thermo Scientific).

IRP activity measurement

K562 cells were used to perform IRP activity measurement. Cells were cultured in the same conditions as for intracellular iron measurements with an increase dose of DFX. Total IRP RNA-binding activity was measured by electrophoretic mobility shift assays with 5 µg of total protein extracts. The minimal sequence of human ferritin H-chain Iron Responsive Element (IRE) was biotin-labeled with biotinylated cytidine (bis) phosphate using T4 RNA ligase (Thermo Scientific). The IRE-IRP reaction was carried out as previously described and the complexes were separated on non-denaturing 4% PAGE in 0.5X TBE, transferred onto Hybond™N^+ membrane (GE Healthcare) and the biotinylated bands were detected after interaction with the streptavidin-horseradish peroxidase conjugate by the peroxidase substrate for enhanced chemiluminescence (ECL)(GE Life Sciences). Quantitation of the signals was done with the Image J software (v1.47, Wayne Rasband, Research Services Branch, National Institute of Mental Health, Bethesda, Maryland, USA).

Oxidative stress metabolite measurements

Thiobarbituric acid reactive species include products of the oxidative degradation of polyunsaturated fatty acids, in particular MDA. We used the modified method of Ohkawa et al (Ohkawa, Anal Biochem, 1979), based on the reaction of aldehyde functions of MDA released by acid hydrolysis at 95°C with thiobarbituric acid forming a pink-colored complex quantified by fluorimetry. Carbonyl assay is based on the reaction of carbonyl groups in protein with 2,4-dinitrophenylhydrazine to form 2,4-dinitrophenylhydrazone, which was estimated spectrophotometrically at 380 nm after trichloroacetic acid precipitation of proteins. Glutathione peroxidase activity was determined by the modified method of Gunzler using tertbutyl hydroperoxide as substrate (Gunzler, Chem Klin Biochem, 1974).

Total GSH (GSH + GSSG) was determined as described previously (Akerboom, Meth Enzymol, 1981) based on the spectrophotometric evaluation of the reduction rate of 5,50-dithiobis-2-nitrobenzoic acid (DTNB, Sigma, France) into 5-thio-2-nitrobenzoic acid (TNB). Values were determined by comparing the reduction rate against a standard curve of GSH.

Cu-Zn superoxide dismutase activity (Cu-Zn SOD) was determined by monitoring the auto-oxidation of pyrogallol using the Marklund method (Marklund, Eur J Biochem, 1974).

Total SOD activity Corresponding to the activity of SOD1 (Cu-Zn SOD) and SOD2 (Mn SOD) was measured using an assay based on the competition between pyrogallol oxidation by ·O2 and superoxide dismutation by SOD. SOD2 activity was determined by assaying for SOD activity in the presence of sodium cyanide, which selectively inhibits SOD1 but not SOD2. SOD1 activity

was calculated by subtracting SOD2 activity from total SOD activity. The rate of auto-oxidation is taken from the increase in the absorbance at 420 nm.

Ferroptosis investigation

RT-qPCR: RNA was extracted using the *mir* Vana™ miRNA Isolation Kit (Thermo Fisher Scientific) according to the manufacturer's protocol. 500 ng total RNA for each sample was used as input for each reverse transcription reaction, performed using the TaqMan RT kit (Applied Biosystems). Primer for IREB2, CS, ATP5G3, EMC2, ASCF2, RPL8 and GAPDH come from commercial solutions (Thermo Fisher Scientific). Quantitative PCR reactions were performed using TaqMan® Universal Master Mix II, no UNG (life technologies). Triplicate samples per condition were analyzed on a Stratagene Mx3000p qPCR instrument (Agilent Technologies). Differences in mRNA levels compared to GAPDH internal reference control were computed between control and experimental conditions using the ΔΔCt method.

Thioredoxin 1 and 2 knock down cell lines production

K562 cells were transfected by electroporation (Amaxa®Cell Line Nucleofector® Kit V K562, Lonza, Cologne) with Nucleofector II (Amaxa Biosystem, Lonza) with three different pEBV-based plasmids expressing shRNA sequences: one with an inefficient shRNA sequence widely documented in the literature and used as a control (CTL), one targeting the TRX1 gene (NM_003329; nucleotides 208 to 226) and one against TRX2 (NM_012473, nucleotides 195 to 213). These replicative pEBV-based plasmids imposed a stable and efficient knock down, as reported in the literature for numerous genes (Biard, Nucleic Acids Res, 2007). konck down cells were called $TRX1^{KD}$ and $TRX2^{KD}$ cells. All plasmids contain a resistance gene against hygromycin. Knock down phenotype was checked by Western blotting analyses. Twenty to forty µg of total proteins were resolved by sodium dodecyl sulfate-polyacrylamide gel electrophoresis on 8% gels, and proteins were transferred to polyvinylidene difluoride membranes. The blots were saturated with 2% bovine serum albumin in TBS-Tween 0.2% and probed overnight at 4°C with antibodies against Trx1 (1:200, Santa Cruz), Trx2 (1:1000, Cell Signaling) or actin (1:250, Sigma Aldrich). Following three washes with TBS-Tween 0.2%, the blots were incubated with peroxidase-coupled goat anti-rabbit IgG (Bethyl) at a dilution of 1:5000 for 1 h at room temperature, followed by detection with the ECL reagent.

NFKB reporter assays

Activation of NF-κB was measured for the K562-Thioredoxin 1 and 2 knock down cell lines. These cells were transfected By nuclear electroporation (Amaxa®Cell Line Nucleofector® Kit V K562, Lonza with Nucleofector II Amaxa Biosystem) of two plasmids: pGL4.32[luc2P/NF-KB-RE/hygro] (ref E849A, Promega, Madison) and pGL4.75[hRluc/CMV] (ref 693A, Promega, Madison). Both plasmids contain a different luciferase activity. The first plasmid displays the firefly luciferase activity under the dependence of a NF-κB responsive element, whereas the second plasmid constitutively produces the renilla luciferase activity and was used as control of transfection. After nuclear electroporation, the cells were cultured in the same iron overloaded medium as described before with DFX 3µM or N-acetyl cysteine 1mM for 48 hours. Then the cells were harvested, washed and the number of cells and their viability was determined with a Luna™ automated cells counter (Logos Biosystems, Korea). Then luciferase activity was measured using the Dual-luciferase® reporter assay system (Promega, Madison) on a CLARIOstar plate reader (BMG Labtech, Ortenberg, Germany) following the manufacturer's protocol. Each condition was read in duplicate. For each condition, the firefly luciferase activity was reported to the Renilla luciferase activity and the data were normalized with the number of cells.

Next generation sequencing

Samples were studied by high-throughput sequencing (HTS) of 36 genes recurrently mutated in myeloid malignancies. This included genes encoding proteins involved in signal transduction (CBL [exons 8-9], CSF3R [exons 14-18], FLT3 [exon 20], JAK2 [exons 12 + 14], KIT [exons 8-11 + 17], KRAS [exons 2-3], MPL [exon 10], NRAS [exons 2-3], PTPN11 [exons 3 + 13], RIT1 [exon 5], SETBP1 [exon 4]), transcription (BCOR [exons 2-15], BCORL1 [exons 1-12], ETV6 [exons 1-8], GATA2 [exons 2-6], RUNX1 [exons 3-8]), chromatin modification (ASXL1 [exons 11-12], EZH2 [exons 2-20], DNA methylation (DNMT3A [exons 2-23], IDH1 [exon 4], IDH2 [exon 4], TET2 [exons 3-11]), RNA splicing (SF3B1 [exons 13-16], SRSF2 [exon 1], U2AF1 [exons 2 + 6], ZRSR2 [exons 1-11]), cohesin complex (NIPBL [exons 2-47], RAD21 [exons 2-14], SMC1A [exons 1-25], SMC3 [exons 1-29], STAG2 [exons 3-35]), tumor suppression (PHF6 [exons 2-10], TP53 [exons 3-11], WT1 [exons 7 + 9]] and other pathways [CALR [exon 9], NPM1 [exon 11]). Libraries were prepared using Ampliseq™ System according to the manufacturer's instructions and run on sequencing on Personal Genome Machine® (PGM,

Life Technologies®). A high depth of coverage (>2000X) was obtained for all genes. Data were processed by Torrent Browser (Life Technologies®) and SeqNext (JSI Medical System®). Frameshift and nonsense variants were always considered as relevant mutations. Single nucleotide variants were retained in the absence of description into public databases of human polymorphisms and effects on protein function were predicted with established prediction tools (SIFT, PolyPhen-1, PolyPhen-2, MAPP, PhD-SNP and SNAP). Notably, because of technical limitations, the mutation c.1934dupG in ASXL1 cannot be detected with PGM sequencing justifying its systematic validation by Sanger sequencing as previously described (Duployez, Blood, 2016).

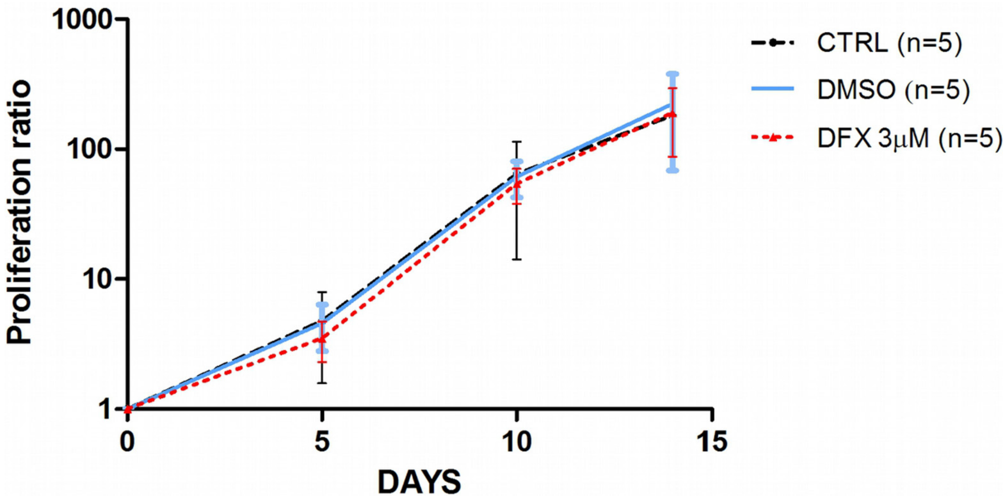

Supplementary Figure 1: Effect of low dose of deferasirox on 5 healthy donors CD34$^+$ cells. Proliferation rate of CD34$^+$ cells from healthy donors (n=5) treated with low dose of DFX in our erythroid differentiation model. There was no effect of DFX 3μM suggesting a specific role of low dose of deferasirox only on MDS samples.

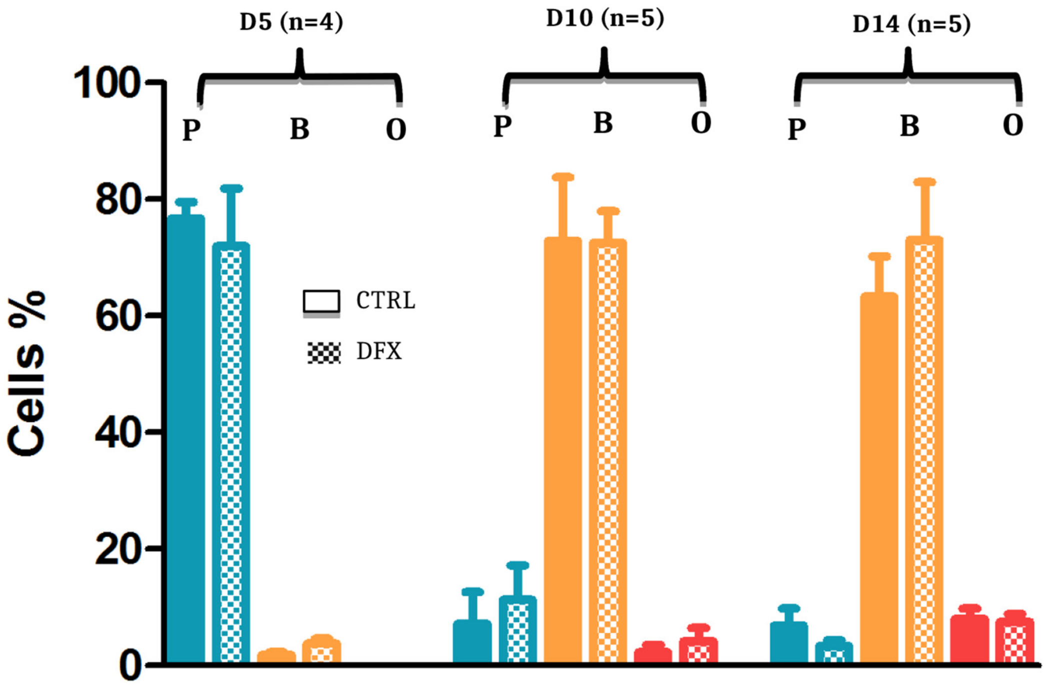

Supplementary Figure 2: Effects of low dose DFX on erythroid differentiation. Global erythroid differentiation was studied by flow cytometry by monitoring three populations of cells: proerythroblasts (P) CD71^{+}/CD235^{-}, basophil erythroblasts (B) CD71^{+}/CD235^{+} and orthochromatic erythroblasts (O) CD71^{-}/CD235^{+} (filled bars: control cells; dotted bars: DFX-treated cells).

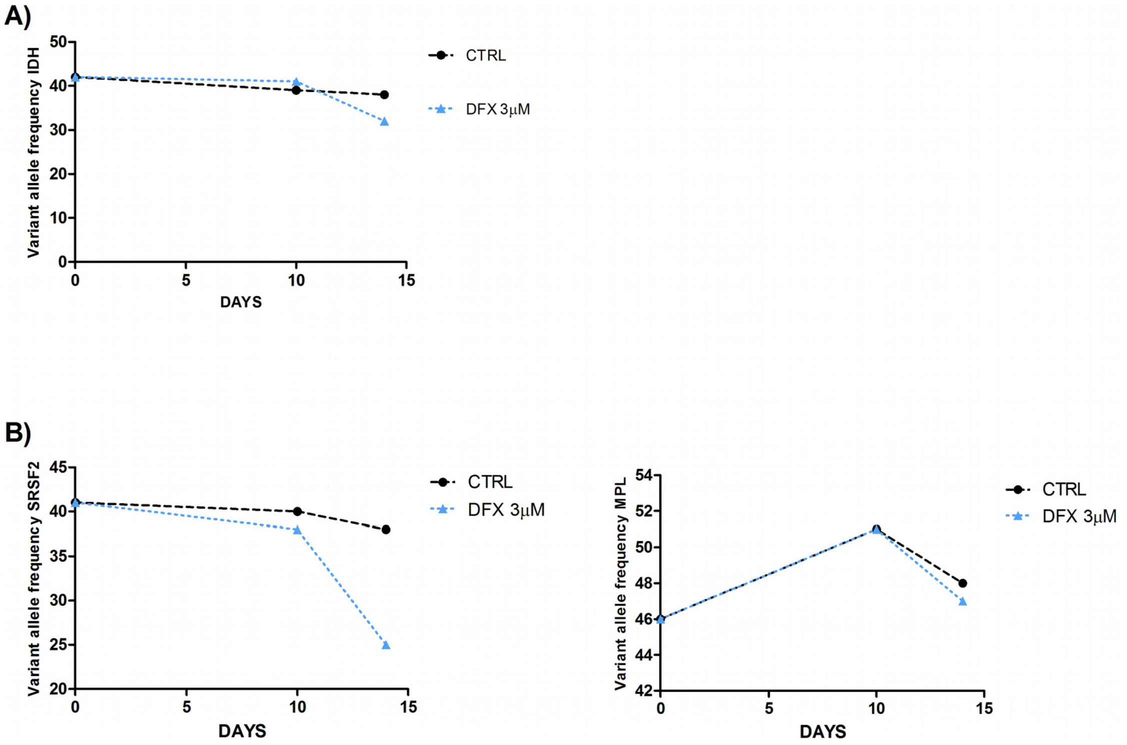

Supplementary Figure 3: Evolution of variant allele frequency. Evolution of variant allele frequency (VAF) concerning CD34$^+$ cells from 2 MDS patients. We applied the erythroid differentiation model and we have followed the VAF. The first patient harboring IDH1 gene mutation **(A)** and the second patient **(B)** two gene mutations: one in SRSF2 gene and the other in MPL gene.

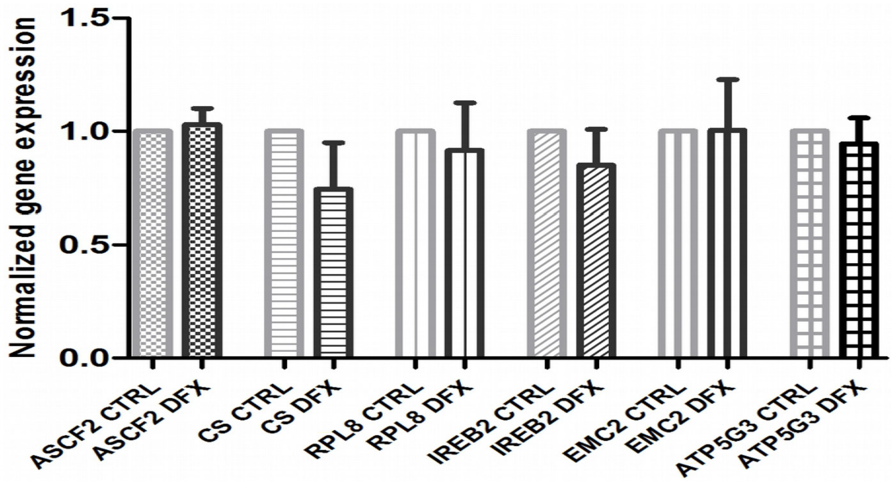

Supplementary Figure 4: Ferroptosis investigation. Relative gene expression by RTqPCR for 6 genes known to be specifically activated in ferroptosis process (RPL8, IREB2, ATP5G3, CS, ASCF2 and EMC2) from 7 samples treated or not with DFX 3µM (p=ns).
